# SARS – CoV 2 imapct’s on menthal health. Case study, psychiatric hospitals “Ali Mihali”, Vlorë

**DOI:** 10.1192/j.eurpsy.2021.1789

**Published:** 2021-08-13

**Authors:** E. Shaska

**Affiliations:** Acute Service, Psychiatric Hospital “Ali Mihali” Vlorë, Vlorë, Albania

**Keywords:** Menthal Disorder, Menthal Health, Patients, SARS CoV2

## Abstract

**Introduction:**

The aim of this paper is to analyse the impact of SARS – CoV 2 on Menthal Health. Based on the studies pacients infected with COVID-19 manifest severe menthal health problems during or after infection.

**Objectives:**

How do different people face the acute phase of SARS-CoV-2 infection? How do menthal health problems influence the disease’s trajectory? What kind of the menthal health disorder occur in people status post Covid?

**Methods:**

We have used a regular, clinical strategy involving adults aged 21-61 years infected with SARS-CoV-2. The research was conducted over the period July-December 2020, in 5 patients (3 males and 2 females) hospitalized in the Psychiatric Hospital “Ali Mihali” Vlora. The assessment on the diagnosis was made conforming to the diagnostic criteria of DSM-5 based on structured clinical interview (information from family, friends, etc.) and examination of mental status

**Results:**

According to the studies SARS CoV2 affects with serious problems the Menthal Health. Some of them are: Sleep disorder. Anxiety disorder. Major Depressive Disorder. Bipolar disorder Psychotic disorder

**Conclusions:**

Patients infected with SARS-CoV-2 must be provided with a family physician psychological evaluation during the acute and post-COVID-19 phase. All individs status post COVID-19 who have lost their daily functioning and pose a risk to themselves and others must be recommended to CMHC for multidisciplinary treatment All COVID hospitals and wards must be equipped with multidisciplinary teams (psychiatrist, psychologist, social worker, mental health nurse) and each clinical record must have current mental status assessment and follow-up in case dynamics.

**Disclosure:**

No significant relationships.

